# Discretion and contextual influences on creative thinking

**DOI:** 10.3389/fpsyg.2025.1565741

**Published:** 2025-07-16

**Authors:** Harun Tadik, Mark A. Runco, Kadir Bahar

**Affiliations:** ^1^Department of Educational Psychology, University of Georgia, Athens, GA, United States; ^2^Creativity Research & Programming, Southern Oregon University, Ashland, OR, United States; ^3^Radical Creativity, Aalto University, Helsinki, Finland

**Keywords:** divergent thinking, discretion, creativity, Big Five, originality, ideation

## Abstract

This investigation used a new method to measure the role of discretion in the creative process. In this context, discretion refers to a particular kind of awareness, and more specifically to an individual's intentional engagement in the creative process. In this investigation, discretion was operationalized as decisions about whether or not to express creative ideas in different social contexts. Discretion may lead an individual to express many creative ideas in one setting but fewer or no creative ideas in a different setting. Two sets of divergent thinking and idea preference measures were adapted from previous research and used to assess creative potential, along with self-reports of ideation, Big Five personality traits, and creative attitudes and values. The divergent thinking tasks were presented with instructions that suggested different social contexts. One context was relaxed and playful conditions, which previous research has shown to support originality. The other instructions described a work environment where there was pressure to succeed and an emphasis on correct ideas. Statistical analyses confirmed differences between the two conditions, suggesting that individuals exercised discretion when they generated ideas. Correlational and hierarchical regression analyses indicated that openness to experience, education, and creative potential were significantly related to discretion. Practical implications and suggestions for future research are discussed.

## Discretion and contextual influences on creative thinking

Creativity involves a variety of elements. This is one reason that creativity has sometimes been called a *syndrome* and other times a *complex* (MacKinnon, 1965; Mumford and Gustafson, 1988). It is often good to think divergently, but creative performance also involves attitude and motivation. Certain personality traits (e.g., openness) may come into play. Social context is also influential (Amabile, 1983; Burns et al., 2015; Csikszentmihalyi, 2006; Puccio et al., 2007; Simonton, 2000). Mindful individuals take certain features of the environment into account and then make decisions about whether or not it is wise to express creative ideas. In a sense they are exercising *discretion*. This is the label given to the process whereby individuals weigh particular features of the environment such that they either allocate resources to original efforts or instead decide to rely on routine, habit, and convention (Runco, 1996, 2003). This kind of discretion may be seen as a kind of creative awareness, and it may be necessary for an individual's intentional engagement in the creative process.

Even highly creative individuals may not feel comfortable expressing novel ideas or behaviors in unsupportive environments. In such environments, they may decide that it is best to express only conventional ideas and behaviors. If the context supports creativity, individuals may invest time and energy in developing their creative abilities and are likely to generate and share novel and surprising ideas. Creativity is not something that creative individuals express all of the time; instead, they control their “weirdness” (Barron, 1993). Note that the expression of creativity probably depends on both personal characteristics (e.g., personality or attitudes) and social context. The present investigation examined both of these in an attempt to predict variation in creative behaviors and ideation.

In organizational settings, individuals are likely to share creative ideas when risk-taking and new ideas/perspectives are appreciated (Shalley et al., 2009). By contrast, perceived threat and stress may undermine creative production (Runco, 1996; Walton and Kemmelmeier, 2012). The meta-analysis of Hunter et al. (2007) summarized the climate that best predicted creative performance. Challenge, resources, autonomy, management support, flexibility, and risk-taking, had sizable effects. Studies in school settings have also confirmed that creative behavior varies significantly in different social contexts (Runco et al., 2017, 2016). Runco et al. (2017) demonstrated that students are likely to express more creativity outside of school, which is relatively free from authority figures, pressure, and stress, compared to school settings. All of these confirm that there is variability in individual creativity in different social contexts.

Individual differences are also important determinants whether or not creative ideas will be expressed. The Big Five personality framework which describes extraversion, agreeableness, conscientiousness, neuroticism, and openness to experience (Costa and McCrae, 1985), offers one explanation. Individuals who are high in agreeableness and conscientiousness are unlikely to choose or seek unconventional ideas or violate social and organizational expectations. People who are high in extraversion and openness to experience, on the other hand, challenge convention more easily, experiment with new ideas and approaches, and go against the crowd, following their intrinsic interests (Feist, 1998; Karwowski et al., 2013; Karwowski and Lebuda, 2016; Martinsen, 2011; Puryear et al., 2017; Sung and Choi, 2009). Regarding introversion-extraversion distinction, it can be concluded that people with introversion tendencies are more likely to be sensitive to contextual stimuli. Ideational tendencies (Plucker et al., 2006) and attitudes and values (Basadur et al., 2000) may also influence the degree to which contexts influence creative expression.

### Rationale and research questions

The present study was designed to examine both social context and personal characteristics. It is unique in its use of a new method for measuring the discretion that is apparent when individuals express their creativity. More specifically, this investigation explored the relationships among discretion, creative potential, the Big Five personality traits, and attitudes and values about creativity. A social context that is perceived to encourage creativity, novel ideas, and risk-taking was expected to foster creativity. On the other hand, a social context that involves judgments, threats, stress, and underestimation of creativity was expected to discourage creative ideation (Amabile et al., 1996; Runco, 1996; Shalley and Gilson, 2004; Walton and Kemmelmeier, 2012; Zhou and George, 2001, 2003).

Utilizing both restrictive (work setting) and relaxed (playful) social contexts, this study used a unique method to measure discretion, which here refers to changes in expression of creativity attributable to contextual differences. Then, the relationship of discretion with other variables was examined to explore which individuals are more vulnerable to influences of contextual characteristics and which are more likely to express creativity regardless of contextual differences. This study was designed to answer the following research questions:

1. Is the new discretion method adequately reliable?
2. Do different social contexts elicit significantly different fluency, originality, and idea preference scores?
3. Is discretion related to age, gender, and educational level?
4. Is discretion related to the Big Five personality traits, attitudes and values about creativity, and creative ideational tendencies?

## Method

### Participants

The sample included 200 (93 male and 107 female) Amazon Mechanical Turk (MTurk) respondents. MTurk is an online platform for data collection, and its use has become increasingly popular. It provides heterogeneous samples and does not rely on college students. Hauser and Schwarz (2016) indicated that MTurk participants were more attentive to the given measures than college students and showed larger change scores in content and instruction manipulations. The mean age for the sample was 43.89 (*SD* = 11.85). Regarding ethnicity, 82% were White, 9% African American, 4% Asian or Pacific Islander, 4% Hispanic, and 1% others. Forty percent of the participants reported that they had a high school diploma, 48% a bachelor's, 11% a masters, and 1% a PhD degree. The data were collected from individuals in the United States and limited to participants older than 18 years. MTurk offers a highly adaptable platform that permits researchers to establish specific participation criteria. In the present study, participants were included only if they completed all required measures within a single session. Additionally, individuals who completed the tasks in less than 30 min or exceeded 60 min were excluded from the sample to maintain consistency in data collection. An attention-check question was integrated into The Big Five Inventory measure to ensure data quality, and participants who failed to respond accurately to this question were also excluded from the final sample. To ensure the work experience that could influence the exercise of discretion, the sample was also limited to individuals who had been employed for the last 3 months. The need for work experience will be clearer as details of the procedure are presented.

### Procedure

Following the consent form, the participants were asked to report their demographic information, specifically age, gender, educational level, and whether they were employed for the past 3 months. Then they completed the measures in the following order: Creative Attitudes and Values, Idea Preference Form B, divergent thinking (DT) Titles with work setting instructions, Runco Ideational Behavior Scale (RIBS), Idea Preference Form A, DT Titles with relaxed (party) instructions, and the Big Five personality measure. The participants completed the measures in one session. The mean time taking to complete the measures was 34 min.

### Measures

#### Idea preference

Idea Preference measures have been used to assess judgment accuracy (or called idea evaluation) skill (Blair and Mumford, 2007; Rietzschel et al., 2014), which is an important component of creative thinking (Runco and Smith, 1992). The measures were included in the study based upon the assumption that people's preferences for unusual ideas may change depending upon the social context. Two Idea Preference measures were adapted from Runco and Charles (1993) and modified for this study. Both forms included a list of 20 responses to a Titles DT task. Form A lists 20 alternative titles for the movie *The Lord of the Rings* and asks participants to rate each response to indicate how much they like each idea. It uses a game-like setting with the following instructions:

*Suppose you have a party and only your good friends (not family or co-workers) are there. An hour into the party, one of your friends starts a conversation about the most popular movies of the last 20 years. She claims that the producer of The Lord of the Rings could have chosen a much better title for this movie. She gives the following alternative titles as examples of alternative titles for The Lord of the Rings. Some of your other friends do not agree with her, and so she asks everyone to rate each of the following alternative titles. Go ahead and rate the alternative titles using your own judgment. Give high ratings to titles you like and low ratings to titles you do not like. Remember that you are among good friends. Have fun*.

Form B had the same design with a different set of 20 responses for the movie, *Harry Potter*. In contrast to Form A, Form B uses a work setting scenario with the following instructions:

*Suppose you have been working for a film production company over the last 3 months and are still in the probation period. Your company has just produced the movie, Harry Potter, and is now ready to release it. Your supervisor is in charge of determining the best possible titles for movies before they are released, and she has just decided to change the title, Harry Potter. She will use one of the following alternative titles for this movie and has asked you to rate each of those alternative titles based on your own judgment. Your contract will be renewed for the next year or terminated immediately based on the results of this final probation-period task. Given this critical situation concerning your job, rate each of the following ideas based on your own judgment*.

For each form, 10 original and 10 unoriginal responses were adopted from a previous study. Original responses were unique responses in the previous sample. Unoriginal ideas were expressed more than 10 times in the previous sample. Both forms were answered on a 5-point Likert scale with the following response options: *(1) I don't like this idea at all, (2) I dislike this idea a little, (3) I neither like nor dislike this idea, (4) I like this idea a little, (5) I think this is an excellent idea*. The order was reversed for unoriginal ideas, and a cumulative score was calculated across the items. High scores indicated a greater preference for creative ideas. It is important to highlight that the Titles tasks were not counterbalanced across the two experimental conditions (work vs. play). Instead, the two tasks were randomly assigned to either work or party conditions. Given that both films, “The Lord of the Rings” and “Harry Potter,” belong to the fantasy genre, it is assumed that neither would inherently confer an advantage within a work or party setting.

#### Divergent thinking

Two sets of DT Alternative Titles test (Form A and Form B) were used to assess creative potential and calculate the discretion scores. DT Titles tests were adapted originally from Guilford (1968). The version used here asked for alternative titles for given movies, books, plays, and so on. Form A has a party setting scenario and was designed similar to a game-like setting (Wallach and Kogan, 1965). Form A asks for alternative names for the Eiffel Tower, the Statue of Liberty, and the Tower of Pisa. Sample instructions were as follows:

*Suppose you had a party and only your good friends (not family or co-workers) are there. An hour into the party, one of your friends starts a conversation about popular architectural structures. She claims that the Eiffel Tower could have a much better name. She suggests some alternative names for the Eiffel Tower and asks you all to play a game. You will have 3 min and all you need to do is to suggest alternative names for the Eiffel Tower. Remember that you are among good friends… so it is not for competition or judgment. Have fun and list as many ideas as you can*.

Form B had a work setting scenario and again asked for alternative titles. Rather than a game-like context, the tasks were presented as part of a job where performance would affect employment status. Form B asked for alternative titles for the movies *Titanic, Superman*, and *Star Wars*. Sample instructions were as follows:

*Suppose you have been working for a film production company over the last 3 months and are still in a probation employment period. Your company has just produced the movie, Titanic and is now ready to release it. Your supervisor is in charge of determining the best possible titles for movies before they are released, and she has just decided to change the title Titanic. Now, she asks you to suggest alternative titles for the movie…one of them may be used as the new title of the movie. Your contract will be renewed for the next year or terminated immediately based only upon the results of this final probation-period task. Given this critical situation concerning your job, suggest alternative titles for the movie Titanic. You will have 3 min for this task. Use your time wisely and list as many alternatives as you can*.

The responses for each DT task were scored for fluency and originality. Fluency refers to the number of valid responses each participant provided, and originality was calculated based upon a 5% cut-off point. Any response given by 5% of the sample (or less) was rated as original. For relatively large samples, scoring for originality is preferred over uniqueness, which refers to number of responses given by only one person in the entire sample (Reiter-Palmon et al., 2019). A subset of responses was scored by a different rater who had experience in DT scoring and creativity research, as a check of inter-rater reliability. The second rater scored the DT tasks independently and was blind to the social context conditions and the overall research design. The inter-rater reliability for originality ranged from 0.79 to 0.91 for the six DT tasks.

Fluency and originality scores were used in analyses but also a part of the calculation of DT discretion scores. These represented the difference between the two instructional conditions, the assumption being that individuals with effective discretion would have larger discrepancies between the party and work conditions. To give a hypothetical example, an individual might exercise some discretion in the work setting, for example, and only share thoughts that were on task and consistent with demands of employment. That same individual might exercise discretion by discerning that the party setting was more relaxed, and ideas could be playful and unusual.

For some analyses (see Results, below) a uniqueness score was used. Uniqueness is another, slightly more stringent method for estimating originality (Runco and Albert, 1985). It was calculated for each participant and based on responses that had been given by only one person in the sample. The inter-rater reliability for uniqueness ranged from 0.82 to −0.90 for the six tasks.

#### The Runco Ideational Behavior Scale

The RIBS was developed by Runco et al. (2001) to address the criterion problem in creativity research. It measures creative ideation and treats ideas as creative products. The RIBS is a self-report about ideation in the natural environment and thus complements DT tests. This investigation used the 26-item version, a sample of which is: “I come up with a lot of ideas or solutions to problems.” Previous studies have reported a good reliability score for the RIBS (Runco et al., 2001; Plucker et al., 2006).

#### Creative attitudes and values

This measure included 10 descriptive statements that asked participants to use a Likert scale to indicate the level of agreement with each. The statements (e.g., “When solving problems, it is often beneficial to postpone judgment about possible solutions”) are related to attitudes and values associated with creativity. The measure has been found to be reliable (Runco et al., 2022) and correlated positively with divergent thinking (Acar and Runco, 2014). It uses a five-point Likert scale with the following response options: *totally disagree*; *mostly disagree*; *neutral*; *mostly agree*; and *totally agree*. Two of the items were counter-indicative and scored accordingly.

#### The Big Five Inventory

John et al.'s (1991) BFI was used for the assessment of the Big Five personality traits. Only extraversion, agreeableness, conscientiousness, and openness to experience were included here. As mentioned earlier, these personality traits are more inclined to engage with social context, whereas neuroticism tends to be more personal and is associated with emotional instability (Feist, 1998; Karwowski et al., 2013; Karwowski and Lebuda, 2016; Martinsen, 2011; Puryear et al., 2017; Sung and Choi, 2009). The measure has 36 items, 8 for extraversion, 9 for agreeableness, 9 for conscientiousness, and 10 for openness to experience. Internal consistency coefficients have been reported previously and ranged from 0.79 to 0.83 (John et al., 1991).

Recall here that the questions addressed in the analyses were as follows:

1. Do the new discretion measures have adequate reliability? they were adopted from previous research but never used to assess discretion, so reliability was the first question. cronbach's alpha was used for the DT tests. A split-half reliability coefficient was calculated for the idea preference measures. The measures had 20 items, 10 original and 10 unoriginal; thus, each half had 5 original and 5 unoriginal ideas.
2. The primary question concerned the impact of social context. is there a significant difference influency, originality, and idea preference scores for two different social contexts?
3. Is there a relationship between discretion and age, gender, and educational level?
4. Are Big Five personality traits, creative attitudes and values, and ideation related to discretion in creativity?

Three discretion scores were calculated for the third and fourth research questions. The first score of these was derived from the Idea Preference measure. A difference score was calculated using linear regression (Cohen and Cohen, 1983). It provides residuals which represent variance not explained by the Idea Preferences given for the work context. The same procedure was used for the discretion in fluency and originality scores. This method gave us a discretion score which indicated variation in DT and Idea Preference that depended upon differences in the social contexts.

## Results

### Descriptive statistics and reliability of the measures

The participants generated 10,843 responses for the six DT stimuli, 1904 for *Titanic*, 1920 for *Superman*, 1821 for *Star Wars*, 1892 for the Statue of Liberty, 1622 for the Tower of Pisa, and 1684 for the Eiffel Tower task. Descriptive statistics for the six DT tasks, as well as composite scores for fluency, originality, and uniqueness across the tasks, are presented in Table 1. The reliability coefficient for the work setting tasks was 0.91 for fluency and 0.83 for originality. For the party setting tasks, the reliability coefficient was 0.92 for fluency and 0.88 for originality. The overall reliability coefficients across the DT tasks were 0.95 for fluency, 0.91 for originality, and 0.86 for uniqueness.

**Table 1:** Descriptive statistics for the divergent thinking tasks.

	**N**	**Min**	**Max**	***M***	***SD***
*Titanic* fluency	200	1	20	9.15	4.35
*Titanic* originality	200	0	18	6.26	3.80
*Superman* fluency	199	1	20	9.39	4.34
*Superman* originality	199	0	16	6.01	3.49
*Star Wars* fluency	198	2	20	9.02	4.26
*Star Wars* originality	198	0	19	6.70	3.84
Statue of Liberty fluency	198	2	20	9.52	4.24
Statue of Liberty originality	198	0	19	6.48	3.81
Tower of Pisa fluency	198	1	20	8.16	3.95
Tower of Pisa originality	198	0	18	5.21	3.45
Eiffel Tower fluency	196	1	20	8.55	4.30
Eiffel Tower originality	196	0	19	6.38	3.83
Fluency total	199	13	118	53.52	22.53
Originality total	199	4	98	36.85	18.50
Uniqueness total	199	0	58	14.63	10.90

Descriptive statistics for the remaining variables, including Idea Preference, Creative Attitudes and Values (CA&V), Runco Ideational Behavior Scale (RIBS), and Big Five traits, are presented in Table 2. The CA&V's inter-item reliability coefficient was initially 0.59. It improved to 0.70 when item 4 (*Good insights often result from concentrating on a problem. It is best not to take time off when immersed in a project*) was removed. The nine-item version was used in the subsequent analyses. Inter-item reliability was 0.91 for RIBS, 0.90 for Extraversion, 0.86 for Agreeableness, 0.86 for Conscientiousness, and 0.87 for Openness.

**Table 2:** Descriptive statistics for idea preference, CA&V, RIBS, and big five traits.

	**N**	**Min**	**Max**	***M***	***SD***
Idea Preference-HP	200	1.95	3.50	2.79	0.28
Idea Preference-LoR	200	2.05	3.65	2.74	0.27
CA&V	200	2.56	5.00	4.09	0.44
RIBS	200	0.31	3.69	1.69	0.60
Extraversion	200	1.13	5.00	2.93	0.97
Agreeableness	200	2.00	5.00	3.82	0.73
Openness to experience	200	1.20	5.00	3.60	0.76
Conscientiousness	200	1.89	5.00	4.07	0.65

HP, Harry Potter; LoR, Lord of the Rings; CA&V, creative attitudes & values; RIBS, Runco ideational behavior scale.

As discussed before, split-half reliability was conducted to assess the Idea Preference measures. The correlations between the original and unoriginal sets of responses were calculated. For Idea Preference for the movie *Harry Potter*, the correlation was 0.70 for the original set of responses, and 0.66 for unoriginal responses. For *Lord of the Rings* form, the correlation was 0.69 for the original set of responses and 0.64 for the unoriginal responses. In general, reliability coefficients above 0.60 are considered acceptable (Hair et al., 2006). Still, the reliability of Idea Preference should be taken into account when interpreting the results. Overall, the DT tests, RIBS, and Big Five personality traits had excellent reliability, and the reliability coefficients for CA&V and Idea Preference measures were in the acceptable range (George and Mallery, 2003).

### Social context differences on idea preference and divergent thinking

A paired sample *t*-test was conducted to determine if the fluency, originality, and idea preference scores differed in the two social contexts. The results indicated a significant difference between the work and party settings in fluency (*Ms* = 27.52 and 26.01, respectively, *SDs* = 11.82 and 11.73), *t*_(198)_ = 3.10, *p* = 0.002, *d* = −0.13. The results for originality and idea preference were also significant. Participants generated more original responses in the work setting than the party setting (*Ms* = 18.94 and 17.92, respectively, *SDs* = 9.66 and 10.20), *t*_(198)_ = 1.98, *p* = 0.049, *d* = 0.10) and favored original over unoriginal responses in the work setting more than the party setting (*M*s = 2.79 and 2.74, respectively, *SDs* = 0.28 and 0.27), *t*_(199)_ = 1.98, *p* = 0.049, *d* = 0.16).

### Correlational analyses

The relation between discretion in the fluency and originality scores and the personality variables was explored through the correlation analyses. The results are presented in Table 3. It shows that discretion in fluency was correlated positively with openness to experience (*r* = 0.14, *p* = 0.043). However, discretion in originality was not significantly correlated with any of the Big Five personality traits, nor CA&V or creative ideation (RIBS). Discretion in idea preference was related negatively to both the RIBS (*r* = −0.16, *p* = 0.028) and openness (*r* = −0.22, *p* = 0.002). Age, gender, and educational level were not correlated significantly with any of the discretion in creativity variables.

**Table 3:** Correlation between discretion in creativity, big five personality, and creativity variables.

	**1**	**2**	**3**	**4**	**5**	**6**	**7**	**8**	**9**
1. CA&V	-								
2. RIBS	0.20^**^	-							
3. Extraversion	0.10	0.29^**^	-						
4. Agreeableness	0.33^**^	0.12	0.28^**^	-					
5. Openness	0.41^**^	0.52^**^	0.32^**^	0.35^**^	-				
6. Uniqueness	0.16^*^	0.26^**^	0.01	0.08	0.28^**^	-			
7. Discretion in fluency	0.00	0.07	0.01	0.09	0.14^*^	0.23^**^	-		
8. Discretion in originality	−0.02	0.04	−0.02	0.09	0.11	0.28^*^	0.84^**^	-	
9. Discretion in IP	−0.01	−0.16^*^	−0.03	−0.07	−0.22^**^	−0.05	−0.16^*^	−0.11	-

^*^p < 0.05.

^**^p < 0.01.

CA&V, attitudes and values; RIBS, Runco ideational behavior scale; IP, idea preference.

### Predicting discretion in creativity

The creativity variables, CA&V, RIBS, divergent thinking, and the Big Five personality traits were explored further as potential predictors of discretion. One of the main objectives of the study was to determine how contextual differences lead to changes in creative thinking and to determine possible predictors of discretion in creativity. Differences were confirmed by the *t*-test results reported just above. Predictors were examined with regression analyses. These used discretion scores that were calculated following a method described by Cohen and Cohen (1983). Here, scores from the relaxed context were regressed on scores from the work setting. The residuals, as Cohen and Cohen (1983) described it, indicate variances of the relaxed context scores that is not explained by scores from the work setting. Those residuals represent a kind of difference score. The residuals of the regression are typically more reliable than difference scores found by subtracting pre-test from post-test scores. In the present case, this residual variance was indicative of discretion (i.e., the degree to which individuals changed their thinking in response to the setting).

One hierarchical multiple regression analysis was conducted for each discretion score. The first used discretion in fluency as the DV, the next used discretion in originality, and the last discretion in idea preferences. For idea preference, age, gender, and educational level were entered in Step 1. In Step 2, CA&V, RIBS, and creative potential scores (total fluency across the six tasks and total originality across the six tasks) were added. Finally, the Big Five personality traits were added in Step 3. Reviewing the high correlation (*r* = 0.94) between total fluency and the percentage originality scores, and the variance inflation factor (VIF) scores in the regression, multicollinearity was detected. Thus, rather than percentage originality, unique originality scores were used. VIF scores above 10 indicate that the models suffered from multicollinearity (Belsley et al., 1980), and after replacing percentage originality with unique originality, none of the VIF values exceeded 2.27, suggesting that multicollinearity was not a concern.

The regression results are presented in Table 4. Only Step 2 was significant (*R*^2^ = 0.06, *F*_(4, 191)_ = 2.68, *p* = 0.033), indicating that the RIBS and fluency variables were significant predictors of discretion in idea preference. The results indicated that individuals with high ideation, as assessed by the RIBS and DT fluency, were less likely to change their idea preferences in response to social context.

**Table 4:** Multiple hierarchical regression analyses predicting discretion in idea preference.

	**Model 1**			**Model 2**			**Model 3**		

**Predictors**	***B***	***SE***	β	***B***	***SE***	β	***B***	***SE***	β
Constant	0.20	0.32		0.72	0.74		0.65	0.75	
Age	0.00	0.01	−0.02	0.00	0.01	−0.02	0.00	0.01	0.00
Gender	0.01	0.15	0.01	0.02	0.15	0.01	−0.02	0.15	−0.01
Education	−0.07	0.10	−0.05	−0.06	0.10	−0.04	−0.04	0.10	−0.03
CA&V				0.06	0.17	0.02	0.20	0.18	0.09
RIBS				−0.24	0.13	−0.15^*^	−0.13	0.14	−0.08
Fluency total				−0.01	0.00	−25^*^	−0.01	0.00	−0.20
Uniqueness total				0.01	0.01	0.16	0.01	0.01	0.15
Extraversion							0.04	0.08	0.04
Agreeableness							−0.03	0.11	−0.02
Openness							−0.25	0.13	−0.19^*^
Conscientiousness							0.12	0.12	0.08
*R^2^*	0.00			0.06			0.08		
*ΔR^2^*	0.00			0.05			0.03		
*ΔF*	0.24			2.68^*^			1.30		

^*^p < 0.05.

^**^p < 0.01.

CA&V, attitudes and values; RIBS, Runco ideational behavior scale.

For discretion in fluency and originality, demographic variables were entered in Step 1. Because there was a dependency between the discretion variables and fluency and originality scores, only the CA&V variable was used in Step 2. The RIBS was not included, as it was designed as a criterion, not a predictor, of divergent thinking (fluency and originality) (Runco et al., 2001). For the final step, extraversion, agreeableness, openness, and conscientiousness among the Big Five personality traits were added. The results for discretion in fluency are presented in Table 5. Only Step 3 was significant (*R*^2^ = 0.08, *F*_(4, 190)_ = 2.66, *p* = 0.034). It explained an additional 5% of the variance in discretion in fluency. Openness and educational level were marginally significant predictors (*p* = 0.05).

**Table 5:** Multiple hierarchical regression analyses predicting discretion in fluency.

	**Model 1**			**Model 2**			**Model 3**		

**Predictors**	***B***	***SE***	β	***B***	***SE***	β	***B***	***SE***	β
Constant	0.44	0.31		0.37	0.72		0.81	0.78	
Age	0.00	0.01	0.00	0.00	0.01	0.00	0.00	0.01	−0.02
Gender	−0.25	0.14	−0.13	−0.25	0.15	−0.13	−0.24	0.14	−0.12
Education	−0.18	0.10	−0.13	−0.18	0.10	−0.13	−0.20	0.10	−0.14^*^
CA&V				0.02	0.16	0.01	−0.21	0.18	−0.09
Extraversion							−0.04	0.08	−0.04
Agreeableness							0.19	0.11	0.14
Openness							0.24	0.11	0.18^*^
Conscientiousness							−0.22	0.12	−0.14
*R^2^*	0.03			0.03			0.08		
*ΔR^2^*	0.03			0.00			0.05		
*ΔF*	1.90			0.01			2.66^*^		

^*^p < 0.05.

^**^p < 0.01.

A&V, attitudes and values.

The same procedure was repeated for the originality score. None of the models or predictors was significant (see Table 6).

**Table 6:** Multiple hierarchical regression analyses predicting discretion in originality.

	**Model 1**			**Model 2**			**Model 3**		

**Predictors**	***B***	***SE***	β	***B***	***SE***	β	***B***	***SE***	β
Constant	0.11	0.32		0.28	0.73		0.68	0.79	
Age	0.00	0.01	0.06	0.00	0.01	0.06	0.00	0.01	0.04
Gender	−0.11	0.15	−0.05	−0.11	0.15	−0.05	−0.10	0.15	−0.05
Education	−0.15	0.10	−0.11	−0.15	0.10	−0.11	−0.16	0.10	−0.11
A&V				−0.04	0.16	−0.02	−0.24	0.18	−0.10
Extraversion							−0.07	0.08	−0.07
Agreeableness							0.19	0.11	0.14
Openness							0.20	0.11	0.15
Conscientiousness							−0.18	0.12	−0.12
*R^2^*	0.01			0.01			0.06		
*ΔR^2^*	0.01			0.00			0.04		
*ΔF*	0.92			0.06			2.08		

^*^ p < 0.05.

^**^p < 0.01.

A & V, attitudes and values.

## Discussion

In this investigation, three discretion scores, namely discretion in idea preference, discretion with fluency, and discretion with originality, were defined and calculated. They reflect the degree of variation in creativity indices, from context to context. Cronbach's alpha and correlation coefficients indicated that the DT tests were sufficiently reliable, and the idea preference measures demonstrated adequate reliability as well. Having reliability is important and allows meaningful interpretations for further analysis. It was especially important here because this was the first use of the discretion scores. Alternative Titles DT tasks have been used previously (Guilford, 1968), but the version here was new. The structure of the idea preference measures was identical with that one used by Runco and Charles (1993), but the instructions were new in this study.

Consistent with previous research, the current study showed that DT performances vary with context (Amabile et al., 1996; Shalley and Gilson, 2004; Zhou and George, 2001, 2003). The participants generated significantly more ideas and offered more novel responses when told to think about being in a work setting. Similarly, for the idea preference measures, the participants chose original responses over unoriginal and popular ideas significantly more often in work setting. Although a difference in DT performance was expected in two social contexts, the unexpected finding was that the work setting tasks elicited greater fluency, originality, and preference for original responses. Perhaps the work setting motivated the participants more than the party. That is a question for future research.

Blair and Mumford (2007) reported that individuals prefer ideas that are consistent with social norms and avoid risky and original ideas when evaluation and judgment are present. In this respect, the present findings should not come as a surprise. Recall here that the DT test instructions for the work stated that participants could lose their job if they performed poorly. One possible explanation for the current findings is that the task instructions were hypothetical, and thus the socially-oriented context may have exerted only a moderate level of stress. The meta-analysis by Byron et al. (2010) showed that a social context with less evaluation and fewer stressors may increase creative production, and the presence of a certain level of stress may motivate individuals to engage in generating creative responses and focusing on the given tasks with the optimal level of cognitive resources (Andrews and Farris, 1972; Baer and Oldham, 2006; Pelz, 1988). It would be good to extend this line of research with manipulations of actual settings rather than relying on hypothetical settings.

The DT tasks in the party setting did not include any explicit judgment, but they still may not have been free from external pressure, and thus individuals may have felt more comfortable expressing relatively conventional or expected ideas in groups of friends. It is also conceivable that creativity is more likely to be expressed when there is an explicit demand (Runco et al., 2022), which was true of the party setting tasks. Perhaps the participants felt little need to produce unusual ideas.

The differences in originality and idea preference scores were marginally significant. The context differences might be more robust with other tasks. There are socially-oriented DT tests, such as the Realistic Presented DT Problems or DT Social Games. Although the task instructions for the two sets of tasks were quite different, the tasks themselves may have been interpreted as similar to one another. As indicated by Kapoor and an Khan (2019), socially-oriented problems are more effective in fostering creative responses. Therefore, future research might incorporate socially-oriented DT tests and examine the way discretion in creativity is manifested when social context differences are embedded in the tasks rather than the instructions.

### Correlates of discretion in creativity

The correlational analysis demonstrated a positive association between discretion in fluency and openness, suggesting that individuals with the high “openness to experience trait” are more likely to show fluctuations in fluency when contexts change. This too was unexpected, given that people who are open to experience tend to be less concerned about authority and more likely to follow their intrinsic interests (Feist, 1998; Karwowski et al., 2013; Karwowski and Lebuda, 2016; Martinsen, 2011; Puryear et al., 2017; Sung and Choi, 2009). Chen et al. (2019) showed a high correlation between openness and cognitive flexibility, which refers to being aware of alternative options and a capacity to adapt to given conditions (Martin and Rubin, 1995). Therefore, one possible explanation for the result with openness is that it may be easier for individuals with high openness to exercise discretion and express different levels of fluency, depending upon the contextual characteristics.

The results provided limited insight into the discretion in originality, as it was not correlated with any of the variables. The change in originality attributable to contextual changes was not associated with personality traits, creative attitudes and values, nor creative ideation. Research has demonstrated that originality is not free from both contextual characteristics (e.g., challenge, resources, autonomy, risk taking, and time restriction; Hunter et al., 2007; Runco, 1996; Shalley et al., 2009; Walton and Kemmelmeier, 2012) and the types of settings (e.g., school vs. personal life; Runco et al., 2017, 2022). One possible explanation for the discrepancy is that the fluency and originality are highly correlated, yet distinct constructs (Dumas and Dunbar, 2014; Runco and Albert, 1985).

In contrast to the discretion in fluency, discretion in idea preference was negatively correlated with openness to experience as well as creative ideation (RIBS). The correlational results indicated that individuals with high openness and creative ideation were more likely to be consistent in their idea preferences, regardless of contextual differences. These results are consistent with previous studies (Feist, 1998; Karwowski et al., 2013; Karwowski and Lebuda, 2016; Martinsen, 2011; Puryear et al., 2017; Sung and Choi, 2009) which have suggested that individuals who are open to experience are more likely to challenge expectations and follow their intrinsic interests.

### Predictors of discretion in creativity

Three hierarchical regression analyses were used to explore the way the creativity variables predicted the variation in creativity due to different contexts. Regarding idea preference, the results revealed that fluency and creative ideation (RIBS) were the only variables that predicted discretion in idea preference. Both fluency and creative ideation focus on the quantity of ideas and are conceptually overlapping indices (Runco et al., 2001); thus, it should not be surprising to see a similar relation between idea preference and fluency with creative ideation. These results are consistent with the argument that creativity is associated with the violation of expectations (Lachmann, 2006; Tadik and Esener, 2020). Further, the results showed that contextual differences were unlikely to influence individuals with high fluency. Given that individuals with high ideational fluency are better at selecting original ideas (Runco and Smith, 1992; Silvia, 2008), it may be concluded that they are also better in resisting contextual differences and selecting original ideas in different social contexts. Uniqueness, another index of originality, was not an accurate predictor. This may be due to the fact that it is more stringent and included responses given by only one person. This scoring procedure does not seem to account for whether or not a given response challenges social expectations.

The hierarchical regression analysis indicated that educational level and openness to experience were the only variables that explained a significant portion of the variance in discretion in fluency. Educational level emerged as a significant but negative predictor of discretion in fluency, implying that the higher the educational level, the lower the fluctuation in the number of ideas generated across different socially-oriented settings. Simonton (1983) argued that the relation between educational level and creativity is curvilinear, which suggests that education enhances personal creativity to a certain point, but a high level of education may stifle it. A possible reason for this is that higher education requires specialization (Simonton, 1983). Educational level could also be viewed from the perspective of domain knowledge. Domain knowledge is one of the key components of the Componential Theory of Creativity (Amabile, 1983), and some degree of expertise is expected for creative production (Baer, 2015; Csikszentmihalyi, 2006; Weisburg, 1999). Although high domain knowledge has potential benefits for creativity, it may entail a cost in creativity, such that people with expertise rely on their memories and assumptions, which in turn undermine cognitive flexibility (Rubenson and Runco, 1995; Runco, 2014). From a positive perspective, the results imply that having a higher educational degree led individuals to generate ideas more consistently and tolerate the differences in contexts.

A positive relation emerged with respect to openness to experience and discretion in fluency, suggesting that individuals with the openness personality trait are likely to perform differently in idea generation in different social contexts. As noted above, openness is associated with experimenting with unusual ideas and challenging conventional expectations and the status quo (Feist, 1998; Karwowski et al., 2013; Karwowski and Lebuda, 2016; Martinsen, 2011; Puryear et al., 2017; Sung and Choi, 2009); thus, open individuals are not expected to show significant variation in their creative production based upon the implicit or explicit expectations found in social context. Considering the positive vs. negative relation between discretion in fluency and discretion in idea generation with openness, openness to experience should be studied further to explore why it functions differently for different discretion indices, as well as its relation to cognitive flexibility and contextual characteristics.

The regression analysis indicated that personality traits, creative attitudes and values, and demographic variables were not good predictors of discretion in originality. Although the originality scores in the two different social context scenarios were marginally significant, none of the models and variables in the hierarchical multiple regression showed a significant relation. Originality is one of the defining characteristics of creativity, and it has been documented that a variety of external factors influences the generation of original responses or expression of unusual behaviors or ideas (Blair and Mumford, 2007; Eisenberger and Shanock, 2003; Hunter et al., 2007; Runco, 2016; Runco et al., 2005, 2022). A possible explanation is that the scoring procedure used in this study focused on how often an idea is expressed compared to the entire sample, and thus may not reflect the change in the tone or to what extent a given response challenges the social norms and expectations for the given social contexts. When a response was given 5% or less of the whole sample, it was deemed original. Hence, personality traits and creative attitudes may not be good predictors of discretion in originality with the present scoring procedure. Moreover, personality traits and creative attitudes and values may not have a direct effect and may influence discretion in originality through their interaction with creative potential instead, which was not tested in the present study. For example, individuals with low creative potential may be unable to adjust their creative behaviors even though they are open to experiences and value creativity. A further study that focuses on discretion could provide more concrete results on this possibility.

The present findings have implications for the assessment of creativity. Although hypothetical scenarios and social contexts are not very common in assessments of creativity, these findings indicated that differences in social contexts may lead to significant differences in creativity indices, and thus, should be considered when socially-oriented DT tasks are used. Given that discretion in creativity was measured reliably, with further refinements it could be used as a tool for both school and organizational settings to identify individuals who are more vulnerable to the adverse influences of social context or those who are more likely to shift their creativity in a positive way when the social context stimulates creativity.

## Limitations and future research

One limitation of this study reflects the hypothetical nature of the DT tasks. Although the differences in the given social contexts led to a significant variation in the participants' discretion in creativity scores, placing individuals in actual real life (work and party) settings could lead to different performances. The DT tasks were completed in a relatively short amount of time, and this too could have prevented participants from internalizing the social contexts. Future research could experiment with discretion in real-life conditions. Further, the sample in this study consisted predominantly of middle-aged participants with a variety of educational and professional backgrounds; thus, broad generalizations are not warranted.

Moreover, the research survey was completed individually on an online platform with a time restriction on the DT tasks. Given the differences in group vs. individual creative performance (Isaksen and Gaulin, 2005) and the detrimental effect of time restriction on creativity (Acar et al., 2019), future research could investigate different administration procedures, such as group vs. individual settings with no time restriction. It is important to highlight the limitations inherent in data collection via online platforms. These platforms may not effectively engage participants. Therefore, employing alternative methods that offer enhanced attention mitigation strategies could prove beneficial for future research studies.

A confounding effect was possible given the study design, as the Title DT tasks were not counterbalanced across experimental conditions and the Discretion score for Idea Preference was derived from a single task per condition. Note, however, that movie titles used in the Titles tasks were selected from the same fantasy genre. Also note that the two Title tasks were randomly assigned to the work and party conditions and that these tasks served as measures of Idea Preference and did not involve idea generation. Instead, participants were asked to evaluate two sets of previously-generated responses under two socially oriented conditions. Nevertheless, the findings related to the Idea Preference measures should be interpreted with some caution.

As a final caveat, there might be creative potential not reflected in DT responses. DT is not an all-encompassing measure of creative talent (Runco and Acar, 2012), and as reported in Table 1, the maximum number of given responses for the DT tasks was 20. It is possible that the more creative ideas could be found if a participant went beyond 20 ideas.

The new approach to examine the variation in creativity across different social contexts, introduced in this research, may open several avenues for future research. The limitations discussed above could be addressed in future studies. The current results provided evidence that the DT tasks used in the study were reliable. However, completing six different DT tasks in one session was time consuming, and thus, it would be useful to develop new and more efficient methods. Perhaps future research could integrate contextual differences into the tasks themselves. Along the same lines, it might be interesting to include a context-free task and compare it with tasks like those used herein. For now, we can conclude that social context makes a difference, but a selective one.

## Data Availability

The raw data supporting the conclusions of this article will be made available by the authors, without undue reservation.
